# Optimizing Thyroid Nodule Evaluation: AI Integration Into the Thyroid Imaging Reporting and Data System Through AI-Based Ultrasound Image Analysis

**DOI:** 10.7759/cureus.102893

**Published:** 2026-02-03

**Authors:** Haseeb Arif, Hasan Farooq, Muhammad Omer Altaf, Muhammad D Asjad, Hadiya Mian, Talat Waseem

**Affiliations:** 1 General Surgery, Shalamar Hospital, Lahore, PAK; 2 Artificial Intelligence, National University of Computer and Emerging Sciences, Lahore, PAK; 3 Radiology, Shalamar Hospital, Lahore, PAK; 4 Medicine, Shalamar Medical and Dental College, Lahore, PAK

**Keywords:** acr-tirads (american college of radiology-thyroid imaging reporting and data system (ti-rads) criteria, artificial intelligence(ai), deep-learning, thyroid nodules risk of malignancy, ultrasound (u/s), vision–language model

## Abstract

Background

Thyroid nodules are among the most common endocrine abnormalities, with ultrasound serving as the first-line tool for risk stratification. The American College of Radiology Thyroid Imaging Reporting and Data System (ACR-TIRADS) standardizes evaluation but is limited by interobserver variability and the time required for detailed interpretation. Artificial intelligence (AI) offers the opportunity to address these limitations and to automate diagnostic processes and enhance diagnostic accuracy.

Objective

To develop and evaluate a vision-language AI model for ACR-TIRADS-based risk stratification of thyroid nodules on ultrasound.

Methodology

This retrospective study analyzed 1,000 thyroid ultrasound images collected between March 2024 and January 2025, of which 139 met the inclusion criteria. Images were annotated according to ACR-TIRADS features (composition, echogenicity, shape, margins, echogenic foci). A vision-language AI model (LLaVA-Med, Microsoft Research, Redmond, WA) was trained using a two-stage strategy that included domain-specific pretraining and fine-tuning on a curated dataset. Diagnostic performance was assessed as a binary classification: suspicious (TR3-TR5) vs. non-suspicious (TR1-TR2).

Results

The model achieved an accuracy of 67%, sensitivity of 71%, specificity of 53%, and precision of 84.6%. The F1 score is an average of an AI algorithm's precision and recall, used to evaluate the algorithm's predictive performance. Our model achieved an F1 score of 77%, and its performance favored sensitivity, reducing the likelihood of missed malignant nodules, though specificity remained moderate.

Conclusion

The vision-language AI model trained on ACR-TIRADS features demonstrated promising performance in thyroid nodule risk stratification. Its higher sensitivity and explainable outputs reflect its potential as a supportive screening tool in clinical practice, particularly in settings with limited radiological expertise. Further refinement and multi-institutional validation are warranted.

## Introduction

Thyroid nodules are among the most prevalent endocrine abnormalities encountered in clinical practice, with a detection rate of 19-68% in the general population depending on age, sex, and geographic factors [1]. The majority of these nodules are benign, with a small but clinically significant proportion representing malignancy. Therefore, distinguishing between benign and malignant thyroid nodules is a critical step in ensuring appropriate clinical management while avoiding unnecessary invasive procedures.

Ultrasound (US) plays a crucial role in thyroid nodule assessment as it allows for the visualization of key features that are highly predictive of malignancy, such as shape, echogenicity, margins, and vascularity [2,3]. Additionally, it has superior spatial resolution compared to other thyroid imaging modalities such as computed tomography (CT) and magnetic resonance imaging (MRI) [4,5]. The American College of Radiology Thyroid Imaging Reporting and Data System (ACR-TIRADS) and the European Thyroid Association Thyroid Imaging Reporting and Data System (EU-TIRADS) are some of the most common risk stratification systems (RSS) used to classify thyroid nodules based on their risk of malignancy [6,7]. ACR-TIRADS employs a point-based system that assigns scores to specific US features, providing a more detailed and less ambiguous classification compared to EU-TIRADS, which relies on predefined patterns or the number of suspicious features without assigning numeric values [8]. In terms of performance, ACR-TIRADS has shown greater sensitivity and specificity compared to EU-TIRADS in classifying thyroid nodules. Several studies have reported that ACR-TIRADS exhibits higher specificity in categorizing nodules, leading to more accurate risk stratification. For example, a study by Seminati et al. concluded that ACR-TIRADS demonstrated higher specificity than EU-TIRADS in classifying malignant thyroid nodules (59.0% vs. 52.4%, p = 0.0012) [7]. Additionally, Shen et. al. discovered that ACR-TIRADS showed a higher sensitivity in predicting cancer risk in thyroid nodules compared to other systems (0.879 vs. 0.872 EU-TIRADS) [9].

The limitations associated with TIRADS scoring include the potential for variability in interpretation among radiologists and the time-consuming nature of the detailed characterization required by ACR-TIRADS. However, artificial intelligence (AI) can enhance TIRADS scoring by automating the process, reducing human error and fatigue, and bypassing learning gaps. AI algorithms, particularly convolutional neural networks (CNN), can be trained to recognize patterns and features in US images, aiding in more accurate risk stratification [10]. While AI may have lower standalone accuracy for diagnosing thyroid nodules than experienced radiologists, it can significantly improve performance when used to assist radiologists in identifying specific malignant features. By leveraging AI technology to augment TIRADS scoring, healthcare professionals can benefit from more efficient and accurate risk assessment of thyroid nodules [11]. This integration of AI can streamline the implementation of TIRADS systems like the ACR-TIRADS, leading to improved patient outcomes through enhanced sensitivity and specificity in identifying malignant nodules, while reducing unnecessary interventions [12].

Although several AI systems have been evaluated for thyroid nodule risk stratification, their real-world clinical applicability is still evolving. Prior studies have shown that AI models can match or exceed the diagnostic accuracy of junior radiologists in US assessment and can significantly reduce the number of unnecessary fine-needle aspiration cytology (FNAC) procedures in otherwise benign thyroid nodules. However, many existing studies rely on large, proprietary datasets and opaque model architectures that limit generalizability and explainability.

This study aims to address these gaps by curating a high-quality, annotated dataset of thyroid US images with standardized TIRADS-based labels. Additionally, this study implements a vision-language AI model using LLaVA-Med architecture (Microsoft Research, Redmond, WA) trained to classify and explain thyroid nodule risk using ACR-TIRADS criteria.

We evaluate the diagnostic accuracy, sensitivity, and specificity of the aforementioned AI model in differentiating suspicious versus non-suspicious nodules, in addition to exploring its utility as a clinical decision support tool that augments radiologist performance, especially in settings with limited expertise.

By embedding expert-defined image annotations into a multimodal AI training framework, this study aims to create a scalable, explainable, and efficient system for thyroid nodule evaluation. The integration of AI in this domain has the potential not only to improve diagnostic accuracy but also to enhance workflow efficiency and patient outcomes.

## Materials and methods

Study design and dataset

This retrospective study was conducted following approval from the institutional review board (Ref: SMDC-IRB/AL/2024-038). A total of 1,000 thyroid US images were acquired between May 23, 2024, and January 1, 2025, using two high-resolution US machines: the Toshiba/Canon Aplio 400 Platinum Series (Toshiba Medical Systems Corporation, Tochigi-ken, Japan) and Toshiba Xario 100 Platinum (Toshiba Medical Systems Corporation, Tochigi-ken, Japan). Images were extracted from both systems in equal proportion (n = 500 each).

**Figure 1:**
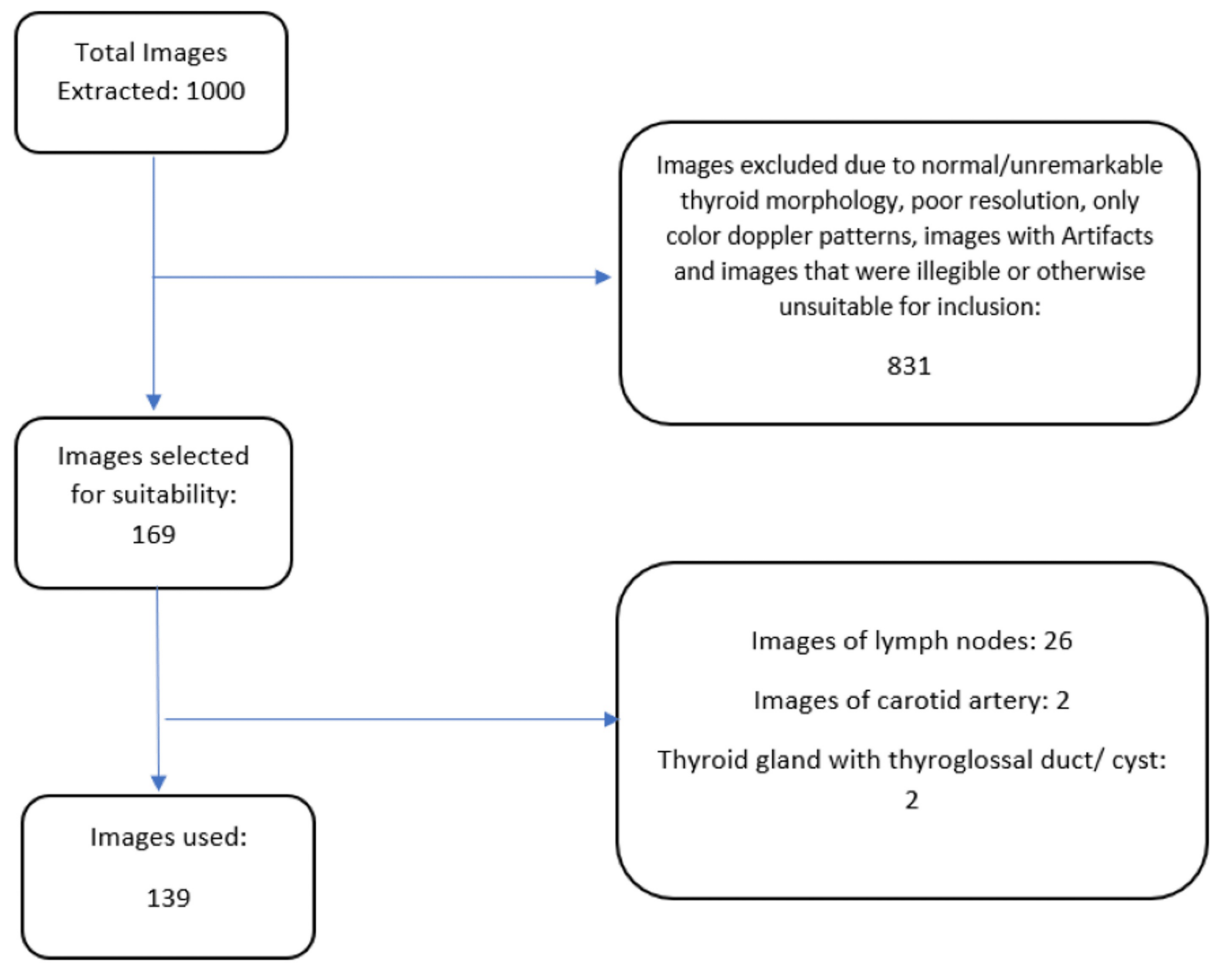
Image selection for AI model training AI, artificial intelligence.

Images were screened for resolution (minimum 1000 × 1000 pixels), clarity, and diagnostic relevance. Exclusion criteria included images with only Doppler views, significant artifacts, non-thyroid anatomical structures (e.g., carotid arteries, lymph nodes), or unremarkable thyroid morphology. After exclusions, 139 thyroid US images were retained for AI model development, training, and validation as shown in Figure 1.

Image annotation

**Figure 2:**
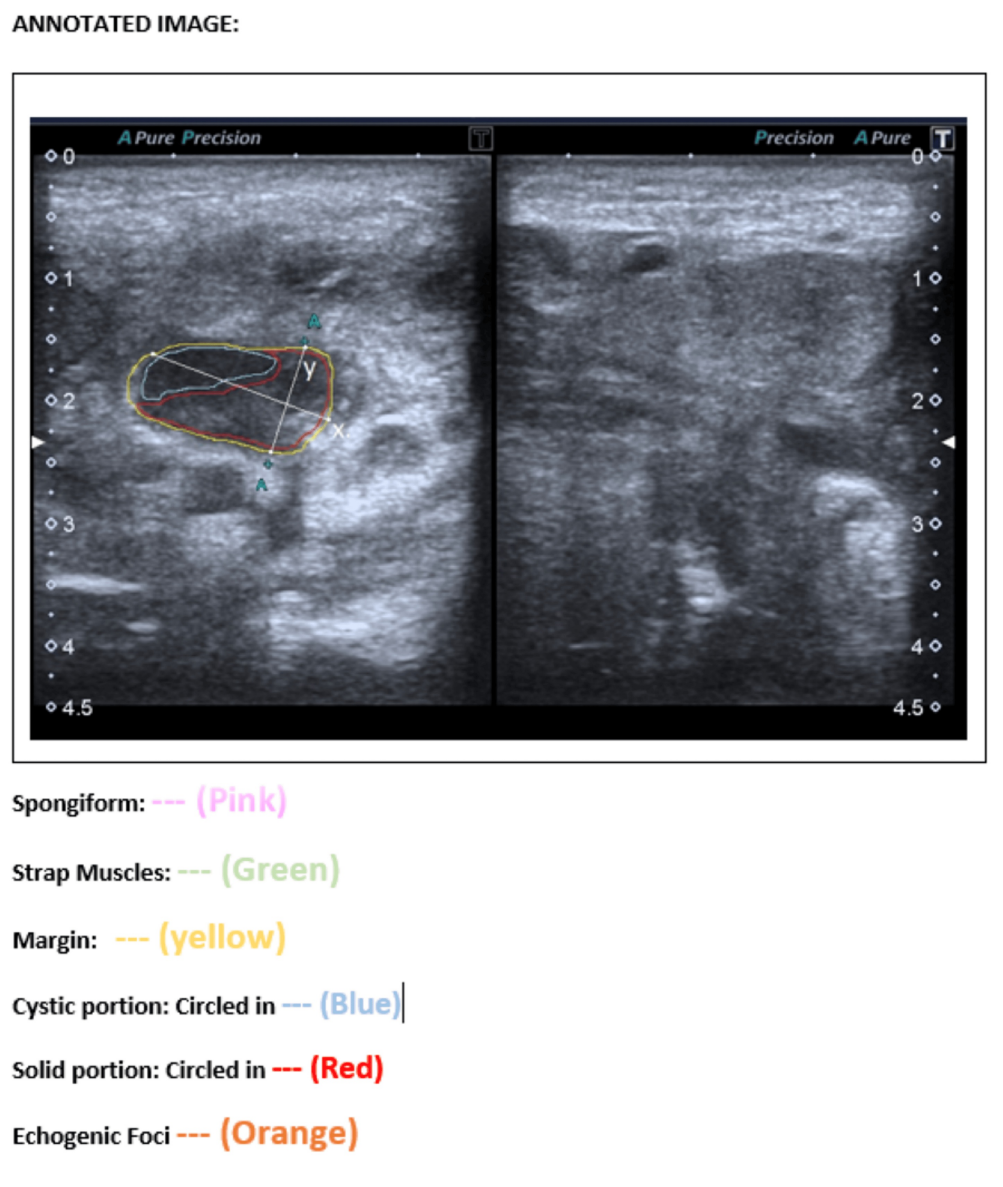
An annotated hypoechoic nodule with mixed solid cystic composition, smooth margin, wider than tall shape, and no echogenic foci. TIRADS score: TR3. TIRADS, Thyroid Imaging Reporting and Data System.

All selected images were annotated using MicroDicom (MicroDicom Ltd, Sofia, Bulgaria) and Microsoft Paint (Microsoft Corporation, Redmond, WA). An example of an annotated thyroid nodule is shown in Figure 2. Annotations followed the ACR-TIRADS lexicon and included five primary features: composition: solid (red), cystic (blue), and spongiform (pink) portions of the nodule were delineated; margins: outlined in yellow; shape: axes were marked and nodules were labeled “wider-than-tall” or “taller-than-wide”; echogenic foci: marked in orange; extra-thyroidal extension: Infiltration into strap muscles was marked in green. An example of an annotated thyroid nodule is shown in Figure 2.

TIRADS classification of images

After obtaining official permission from the American College of Radiology (ACR) to use the TIRADS system, the images were annotated accordingly. Annotations adhere to the ACR TIRADS lexicon, encompassing five principal imaging features: composition, echogenicity, shape, margin characteristics, and echogenic foci. Each feature contributes to a cumulative score used to stratify the malignancy risk of thyroid nodules. A typical annotation example might read: “Composition: Mixed cystic and solid (1 point); Echogenicity: Hypoechoic (2 points); Shape: Wider than tall (0 points); Margin: Lobulated (2 points); Echogenic foci: None (0 points) → Total Score: 5 → TR4 classification”.

**Figure 3:**
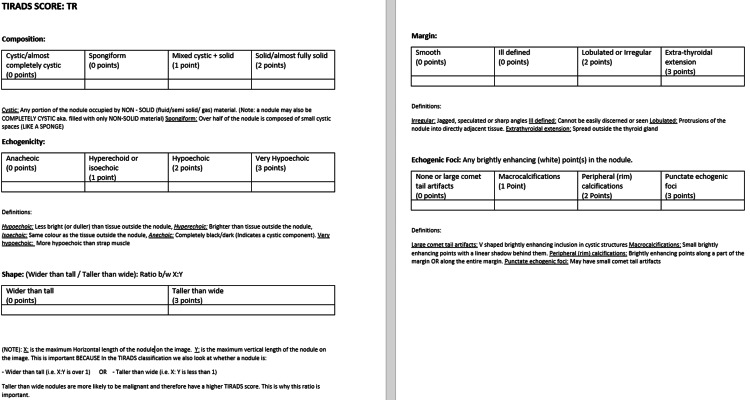
The tabulated scoring system used to score each image after annotation. [6].

This annotation scheme provides high-quality supervision for AI training by embedding domain-specific diagnostic reasoning into natural language form, facilitating multimodal alignment between visual and textual modalities, and supporting explainable model behavior through interpretable intermediate representations. An annotation template is shown in Figure 3.

Data pre-processing

All samples were programmatically converted into a structured JSON format to support training in a visual question answering (VQA) paradigm. Each instance encodes the following: references to both the original and annotated US images, a series of structured clinical questions (e.g., "What is the TIRADS score for this nodule?", "What are the diagnostic features observed?"), and corresponding natural language responses, synthesized from the descriptive annotations. This pre-processing pipeline enables the model to engage in multimodal instruction-following and chain-of-thought reasoning, mimicking human-like diagnostic justification.

Model architecture and training strategy

We employed LLaVA-Med, a vision-language AI model adapted for biomedical imaging tasks, to serve as the backbone for TIRADS classification and reasoning. Due to the limited scale of our task-specific dataset, we adopted a two-stage training strategy to leverage domain knowledge and improve generalizability.

Domain-Specific Pretraining

In the first stage, LLaVA-Med was pretrained on two publicly available medical VQA datasets: "SLAKE" and "VQA-RAD," to establish foundational domain knowledge; this allowed the model to have prior exposure to medical imaging and interpretation, allowing easier subsequent integration of the thyroid US images used in our study.

The VQA dataset "SLAKE" comprises approximately 7,352 image-question-answer triplets across over 1,000 medical images. It spans multiple imaging modalities, including X-ray, CT, MRI, and US, and addresses tasks such as anatomical recognition, abnormality detection, and clinical reasoning. Although the representation of thyroid US is limited, the inclusion of sonographic data enhances transferability to our task domain, which is also related to sonographic or US image interpretation.

The other dataset we used for pretraining was "VQA-RAD," which contains 3,515 question-answer pairs across 315 radiology images, primarily consisting of X-ray, CT, and MRI modalities. Despite the absence of US images, its strong emphasis on diagnostic question framing and structured clinical reasoning provides valuable reasoning and radiological interpretative capabilities. Therefore, this dataset trained the AI model to better understand patterns in diagnostic reasoning from medical imaging. 

Together, these datasets enable the model to learn general visual-textual patterns in radiology, facilitating adaptation to TIRADS-based classification of thyroid nodules.

Task-Specific Fine-Tuning

Following pretraining, the model was fine-tuned on our curated thyroid US dataset using an 80:20 train-validation split. Each training instance consisted of a dual-image input (original and annotated) and a corresponding set of structured questions. The model was optimized to generate both the TIRADS classification and an accompanying rationale, encouraging alignment with clinical diagnostic thought processes.

Evaluation protocol

To facilitate clinically meaningful interpretation, we reformulated the TIRADS scoring task as a binary classification problem, stratifying nodules into two diagnostic categories: non-suspicious: TIRADS TR1 and TR2; and suspicious: TIRADS TR3, TR4, and TR5.

## Results

**Figure 4:**
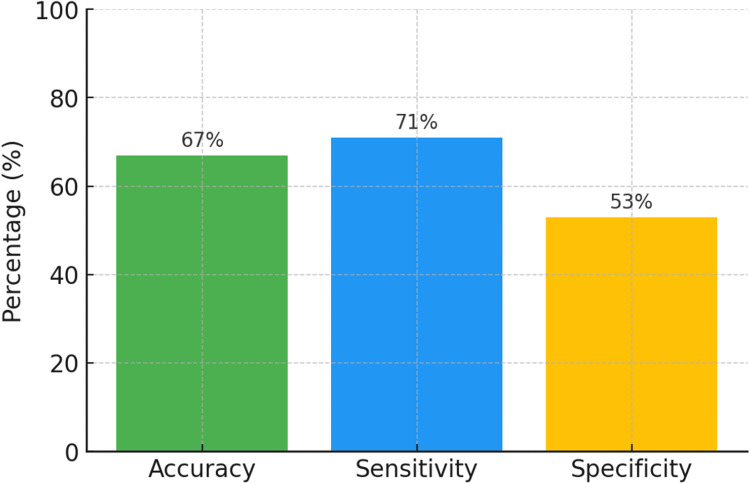
A graphical representation of the AI algorithm's performance metrics.

The model demonstrated a sensitivity of 71%, specificity of 53%, and an overall accuracy of 67% in distinguishing suspicious (TR3-TR5) from non-suspicious (TR1-TR2) thyroid nodules as depicted in Figure 4. This reflects the model’s higher true-positive rate, a critical characteristic for clinical screening tools where missing malignant cases carries significant consequences.

Summary of model performance

The aforementioned results indicate that the model correctly identified 71% of suspicious nodules (TR3-TR5), while maintaining moderate specificity in classifying non-suspicious nodules (TR1-TR2). The results suggest a high true-positive rate, essential in minimizing missed malignancies in clinical screening.

Quantitative performance metrics provide further clarity on these trends. The overall accuracy was 67%, suggesting that approximately two-thirds of predictions were correct. Sensitivity (recall) reached 70.5%, reflecting reasonable proficiency in capturing true positives, whereas specificity remained moderate at 54.5%, underscoring challenges in ruling out negatives. Precision was high at 84.6%, confirming that most predicted positives were indeed correct, and the F1 score of 77% demonstrated a balanced trade-off between precision and recall. This indicates that the model may be particularly valuable in contexts where missing positive cases could have significant consequences, though further refinement is needed to strengthen specificity and overall diagnostic reliability. This matrix further illustrates that the model prioritized sensitivity over specificity, aligning with its screening-focused design.

**Figure 5:**
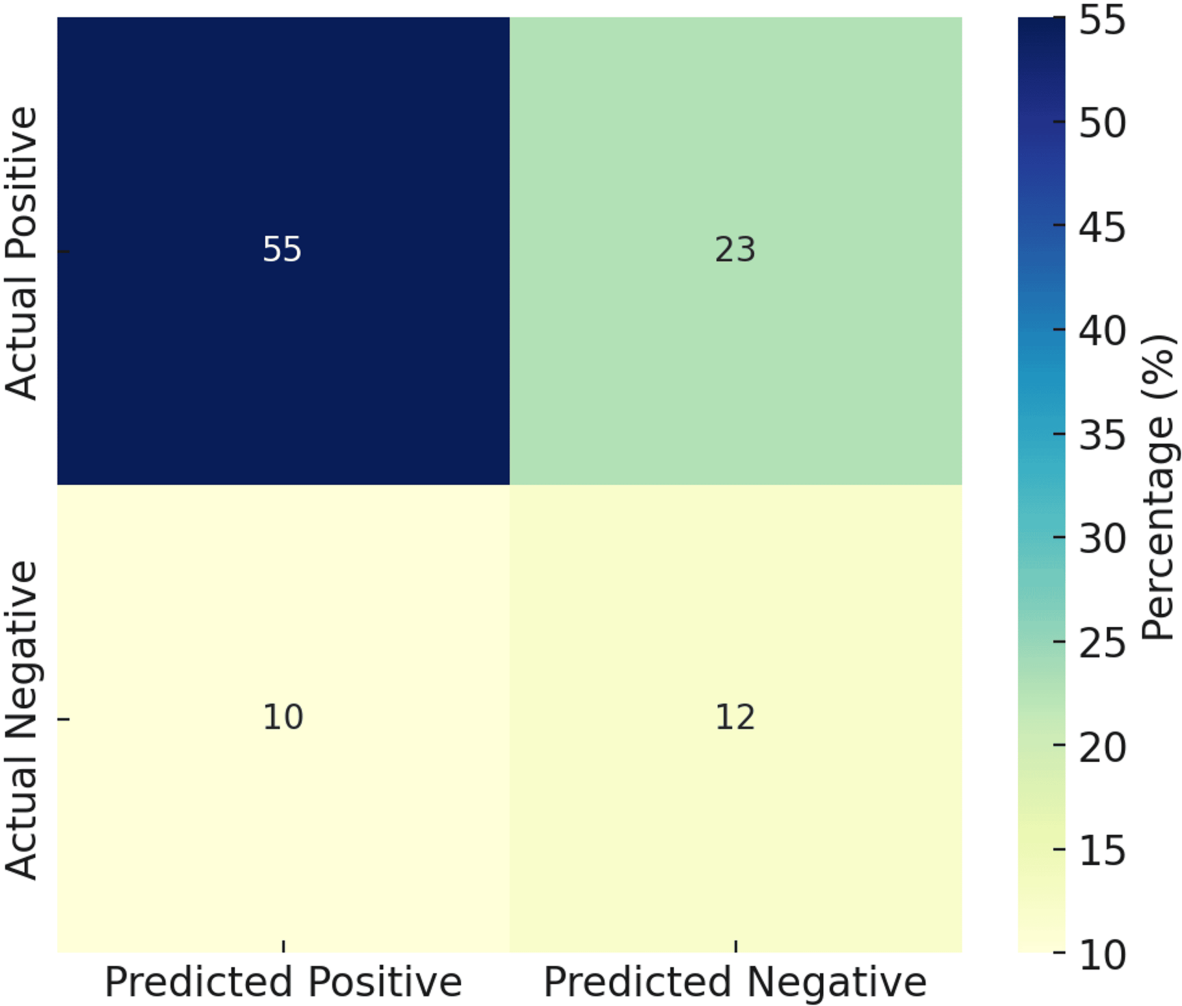
The confusion matrix highlighting a greater ability to identify true positives.

The confusion matrix illustrated in Figure 5 demonstrates that the evaluated model exhibits notable strengths in identifying positive cases, yet reveals limitations in handling negative classifications. Specifically, 55% of predictions were true positives, indicating a superior recognition of actual positive outcomes, while 23% of positive cases were incorrectly classified as negatives, reflecting a considerable false-negative burden. Conversely, only 12% of negative cases were correctly identified as true negatives, with 10% being misclassified as positives. This distribution highlights a model bias toward detecting positives, thereby favoring sensitivity at the expense of specificity.

## Discussion

Improving thyroid nodule assessment through refinement in ultrasonographic evaluation is essential for early detection of thyroid cancer (TC), a highly prevalent malignant pathology. TC is one of the most prevalent malignancies in the world, being described as the second most common malignancy in women in Saudi Arabia and the fifth most common malignancy in American women. Bukhari et al. reported that 15.3% of all reported thyroid lesions over a five-year period at Jinnah Postgraduate Medical Centre in Karachi, Pakistan, were found to be malignant TC [13,14].

The incidence of TC has risen sharply. Studies have shown that TC has been becoming increasingly prevalent in both the USA and Europe, with three out of every 100,000 people developing TC over their lifespan and a greater incidence in women than in men (2-3:1) [15,16]. It was reported that from 1984 to 1985, only nine patients were treated for TC at INMOL hospital, Lahore, Pakistan, whereas 117 patients were treated between 2013 and 2014, exhibiting a 13-fold increase in detection and treatment of TC cases [17]. Nevertheless, the mortality rate for TC has remained relatively stable, with the aforementioned study by Lim et al. reporting an increase of 0.06 per 100,000 people from 1994 to 2013 [15]. Another study found that even though the incidence of TC had increased 2.4-fold from 1973 to 2003, most of it could be attributed to indolent papillary TCs or cancers measuring 1 cm or smaller (49%), causing subclinical disease [18]. This points to endemic "overdiagnosis" and "over treatment" of TCs, further highlighting the importance of the widespread implementation of robust RSS such as the ACR-TIRADS. The ACR TIRADS ensures the avoidance of overtreatment while still adequately identifying clinically significant lesions. Several studies have reported a reduction in unnecessary biopsies by more than half when ACR-TIRADS was used [19,20].

The capability of AI in accurately stratifying thyroid nodules using TIRADS has been thoroughly evaluated in the literature. Chen et al. trained an AI algorithm using 1345 thyroid US images and reported a sensitivity of 83%. In that study, the AI outperformed junior radiologists who had a sensitivity of 70% for accurately assigning TIRADS scores to thyroid nodules [21]. Another study compared the performance of various AI CNNs in stratifying thyroid nodules using ACR-TIRADS. With sensitivities ranging from 85.4% to 91.2%, from the lowest-performing algorithm to the highest-performing algorithm, "Xception" (Google, Mountain View, CA), the AI algorithm, also outperformed three radiologists that they were compared to in that study [22].

The use of AI was also found to significantly improve outcomes when used as an assisting tool for radiologists evaluating thyroid nodules. He et al. evaluated the effect of AI integration on the performance of junior radiologists and discovered that AI integration significantly improved their effectiveness in accurately diagnosing malignant thyroid nodules [23]. Another study trained an AI algorithm, ThyNet, using 18,049 images and found that its clinical integration as a radiologist assistant tool significantly improved the pooled area under the receiver operating characteristic curve (AUROC) from 0.862 to 0.873 (p < 0.0001). Furthermore, it also resulted in a 26.7% reduction in FNAC procedures while improving diagnostic accuracy, indicating an overall reduction in unnecessary FNAC procedures [24]. Similarly, Buda et al. conducted a study developing a deep learning algorithm that provided stratification for malignancy risk. Using 1631 nodules initially, the benign and malignant nodules were then divided into "easy" and "difficult" subsets based on expert consensus. Interestingly, the AI had a higher ROC AUC than radiologists for the "difficult" cases (0.92 vs. 0.70) (p = 0.021) and a similar ROC AUC for the "easy" cases (0.89 vs. 0.92) (p > 0.5). This may indicate that AI can be an indispensable tool for junior radiologists or residents in accurately stratifying and diagnosing complex nodules [12].

Several FDA-approved AI tools for thyroid nodule risk stratification are commercially marketed and readily available for clinical integration and usage. S-Detect 2 is one such deep learning AI-based system, developed by Samsung Medison (Seoul, South Korea) [25]. Having been extensively studied, this tool provides TIRADS-based thyroid nodule risk stratification and a dichotomous benign/malignant prediction. One study that involved 454 thyroid nodules found that S-Detect 2 had a sensitivity on par with that of expert radiologists (97.6% for S-Detect vs 97.6% for radiologists) [26]. Another study found that radiologist sensitivity increased by 8.1% with the assistance of S-Detect 2, while the specificity and accuracy decreased by 28.8% and 14.2%, respectively [27].

AmCAD-UT, a Windows-based software developed by Microsoft (Redmond, WA) in 2018, identifies nodule features and assigns a risk level with a recommendation for FNA or F/U US. Studies have shown similar performance to S-Detect. It also has a sensitivity similar to radiologists (87% vs. 87%), but a lower specificity (68.8% vs 91.2%) and lower AUC (0.72 vs 0.88) [24]. Another study found that AmCAD-UT performed better than radiologists when analyzing intermediate-risk (TIRADS 3 and 4) thyroid nodules; additionally, it was found that the radiologists’ AUC significantly increased with the use of the AI tool (0.728 vs 0.792) (p < 0.0001) [28].

Koios DS (Koios Medical, Inc, Chicago, IL) is a relatively new AI thyroid nodule stratification tool, gaining approval in December 2021. This AI tool can produce nodule descriptors based on the ACR-TIRADS lexicon and provide automated recommendations. In an FDA-led study to evaluate this tool with 500 nodules, the sensitivity for detecting malignancy was found to be 65%, and specificity was found to be 61%; these statistics were reported to be higher than those of other physicians analyzing these images [25]. This is comparable to the performance of the AI tool developed in our study, which had similar sensitivity and specificity. 

Table 1 compares and juxtaposes our model’s performance with existing AI-based systems such as ThyNet, S-Detect 2, AmCAD-UT, and Koios DS. Our model shows comparable sensitivity, though its specificity remains moderate. Importantly, its structure as a vision-language model trained on multimodal inputs offers unique interpretability and alignment with clinical reasoning processes - features that are less emphasized in many black-box AI models currently available.

**Table 1:** A table comparing the performance of our AI model with other commercially available models. [24,25,28].

AI Tool	Sensitivity (%)	Specificity (%)
Our AI Model	71	53
ThyNet	83	78
S-Detect 2	97.6	61-71
AmCAD-UT	87	68.8
Koios DS	65	61

Our study was limited by a lack of availability of multi-institutional data, restricting the generalizability of the AI model. Additionally, due to the operator-dependent interpretability of USG imaging, variations in image acquisition and US technique could limit performance on external datasets, although this was partially mitigated through image annotation. Our study was also limited by a lack of histological confirmation of malignancy, which could be used to better evaluate the performance of the AI model. Additionally, the utilization of a larger, prospective dataset could offer improvement over the current limited retrospective dataset that was utilized for our study. Future studies will encompass larger, more diverse, real-time datasets with direct clinical integration and evaluation of the AI model.

## Conclusions

This study demonstrates the potential utility of a vision-language AI model for thyroid nodule risk stratification based on US imaging. The model, trained on ACR-TIRADS features, performed comparably to other commercially available models despite using a relatively limited dataset. It could offer improved diagnostic performance when used as a support tool for radiologists. Its capacity to generate interpretable, text-based rationales for classification adds value over traditional black-box AI models and aligns with current trends in explainable artificial intelligence (XAI) in medicine.

While the model shows promise as a decision-support tool, particularly in settings with limited radiological expertise, further work is required to improve specificity, expand dataset diversity, and validate the system across institutions. With future refinement, such tools may significantly enhance the accuracy, efficiency, and accessibility of TC diagnostics, contributing to more equitable and consistent care delivery.
